# Acoustofluidic Properties of Polystyrene Microparticles

**DOI:** 10.1021/acs.analchem.3c01156

**Published:** 2023-06-26

**Authors:** Alexander Edthofer, Jakub Novotny, Andreas Lenshof, Thomas Laurell, Thierry Baasch

**Affiliations:** † Department of Biomedical Engineering, Lund University, 223 63 Lund, Sweden; ‡ Department of Bioanalytical Instrumentation, Institute of Analytical Chemistry of the CAS, 602 00 Brno, Czech Republic

## Abstract

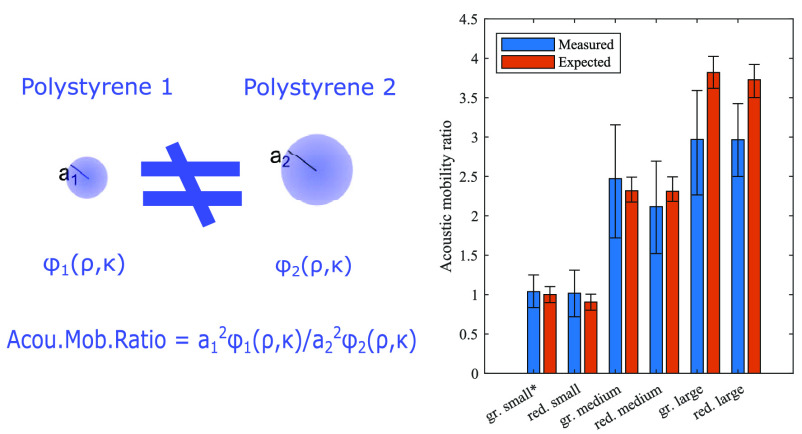

Acoustophoresis has become a powerful tool to separate microparticles and cells, based on their material and biophysical properties, and is gaining popularity in clinical and biomedical research. One major application of acoustophoresis is to measure the compressibility of cells and small organisms, which is related to their contents. The cell compressibility can be extracted from the acoustic mobility, which is the main output of acoustic migration experiments, if the material properties and sizes of reference particles, the size of the cells, and the surrounding medium are known. Accurate methods to measure and calibrate the acoustic energy density in acoustophoresis systems are therefore critical. In this Perspective, polystyrene microparticles have become the most commonly used reference particles in acoustophoresis, due to their similar biophysical properties to cells. We utilized a two-step focusing method to measure the relative acoustic mobility of polystyrene beads of various sizes and colors and present a quantitative analysis of the variation in acousto-mechanical properties of polystyrene microparticles, showing a large spread in their material properties. A variation of more than 25% between different particle types was found. Thus, care is required when relying on polystyrene particles as a reference when characterizing acoustofluidics systems or acousto-mechanical properties of cells.

## Introduction

Acoustophoresis, i.e., the manipulation of micrometer-sized beads, cells, and organisms based on their biomechanical properties by means of acoustic waves, has received attention in the past decade. The technology enables size separation of microparticles,^1^ isolation of circulating tumor cells,^2,3^ and characterization of the mechanical properties of cells^4^ and small organisms such as *C. elegans*.^5^ The acoustic migration velocity of particles or cells under the action of acoustic forces is a function of both the acoustic energy density and the material properties of the specimen.^6,7^ Therefore, accurate methods to measure and calibrate the acoustic energy density in acoustophoresis systems are critical. Typically, this is done by analyzing the trajectories of a reference particle with known density and compressibility.^8^ Due to their similar acousto-mechanical properties to cells, polystyrene microparticles have become the most commonly used reference particles in acoustophoresis. Here, we show that the variation in material properties of polystyrene microparticles is much larger than previously assumed. For example, in 2010, Barnkob et al.^8^ used polystyrene particles to measure the acoustic energy density inside acoustic manipulation devices and thereby characterize the pressure fields. One year later, Hartono et al.^4^ measured the compressibility of red blood cells, MCF-7 breast cancer cells, HEPG2 liver cancer cells, HT-29 colon cancer cells, NIH/3T3 Fibroblasts, and MCF-12A normal breast cells, while using 3–10-μm polystyrene particles as a reference. They chose polystyrene specifically for their known properties. In 2012, Barnkob et al.^9^ used 0.6–10-μm-sized polystyrene particles to characterize the competition between acoustic radiation and streaming forces. Polystyrene particles are employed not only to characterize bulk acoustic wave devices but also to characterize surface acoustic wave (SAW) devices. For example, Ding et al.^10^ used polystyrene particles of many different colors and sizes ranging from 2 μm to 15 μm in diameter to characterize their cell separation application. In more recent works, 15.6-μm polystyrene particles have been used as a reference to measure the compressibility of *C. elegans* by Baasch et al.^5^ Qiu et al.^11^ employed 0.5–4.6-μm particles to assess the competition between streaming and acoustic forces. The most recent acoustofluidic calibration technique, which was published in 2021, uses motile cells to measure the acoustic energy density^12^ and was validated with respect to polystyrene particles. In summary, to characterize acoustofluidic manipulation devices, researchers have almost exclusively relied on polystyrene particles as reference particles.^5,12−18^ The claimed advantages of polystyrene are usually that their material properties are well-known and are independent of their size, color, and manufacturer. One can distinguish between applying polystyrene as a reference for qualitative or quantitative measurements. Qualitative refers to the use of polystyrene particles as ”cell-like” particles because their sizes and mechanical properties are comparable to biological cells. To reduce expenses it can be useful to show the feasibility and optimize the running parameters of an application with polystyrene particles first and then run the final experiments with cells. Even if the material properties of polystyrene are not precisely known, they can be used for such qualitative experiments. In quantitative studies, reference measurements are performed with polystyrene particles, usually to assess the acoustic energy density inside a device. This step is necessary to quantify the performance of a device, the amplitude and shape of a pressure field, or measure the material properties of biological or nonbiological samples. In this study, we investigated the acoustophoretic mobility of differently colored and sized polystyrene particles relative to each other. Ideally, if the material properties of the particles were all equivalent, then the only variation in acoustic mobility would originate from their variation in size. However, we found that different-sized polystyrene particles show a relative variation of more than 25% with respect to their expected acoustic contrast factor, indicating significant differences in their material properties.

## Methods

### Device and Experimental Setup

**Figure 1:**
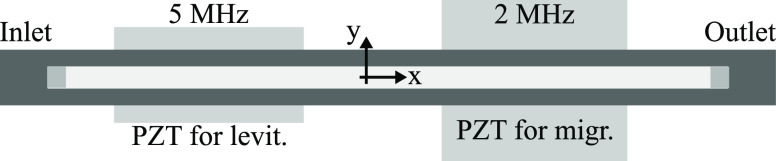
Schematic of the acoustofluidic device. Two piezos were glued to the device to drive both the levitation and the migration modes.

The acoustofluidic levitation and migration device is depicted in Figure 1. The microfluidic channel was DRIE etched 150 μm deep in a silicon wafer (540 μm thick). The channel dimensions were length × width × height = *l* × *w* × *h* = 35.7 mm × 375 μm × 150 μm. The channel was sealed by an anodically bonded glass lid with a thickness of 1130 μm.

Two piezoelectric transducers (Pz26; Meggit Ferroperm Piezoceramics, Kvistgård, Denmark, *l* × *w* × *h* = 12.9 mm × 4.9 mm × 0.4 mm and 10.1 mm × 8.1 mm × 1.0 mm) were glued to the chip (Loctite Super Glue Brush on, Henkel Norden AB, Bromma, Sweden). The connecting wires were soldered to the piezos and connected to function generators (Models AFG 3022B and AFG 3022C, Tektronix, Beaverton, OR, USA) and an amplifier (in-house built). The device was mounted on a round polyoxymethylene (POM) holder (diameter = 110 mm), which had an 80 mm × 30 mm large opening to allow easy access to the device. To mount and secure the device, an aluminum clamp (*l* × *w* × *h* = 42.7 mm × 23 mm × 2.1 mm) with an opening (*l* × *w* = 31.7 mm × 12 mm) was used. A sketch of the device is provided in Section 1 in the Supporting Information.

The driving frequencies of the piezos correspond to the half wavelength modes of the channel height and width and are driving the particle levitation and migration, respectively.

The migration trajectories of fluorescent polystyrene microparticles were recorded by a CMOS camera (Zyla 4.2 scMos; Andor, Belfast, Northern Ireland) mounted to a microscope (DM 2500 microscope, 20×/0.4 objective; Leica Microsystems, Wetzlar, Germany). For fluorescence imaging, a broad spectrum light source (X-cite 120Q; Excelitas Technologies, Göttingen, Germany) was filtered by standard fluorescence filter cubes. One (FITC) had an excitation peak at 490 nm and an emission peak at 525 nm. The other (TRITC) had an excitation peak at 525 nm and an emission peak at 570 nm.

**Figure 2:**
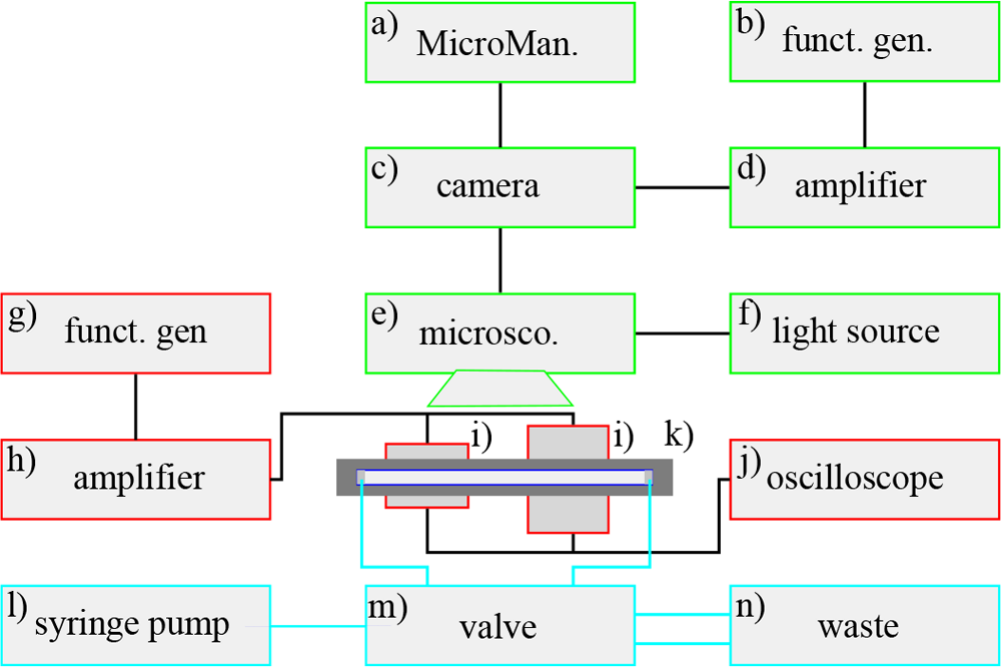
Camera (c) was triggered by an amplified signal (d) from a function generator (b) and delivered the images, which were taken through the microscope (e) and displayed in the Micro-Manager software (a). The piezos (i) were glued to the acoustofluidic chip (k) and actuated by an amplified signal (h) from a function generator (g). The signal to the piezos was monitored by an oscilloscope (j). The fluid flow was driven by a syringe pump (l), regulated by a valve (m), and connected to a waste outlet (n).

The fluid flow was controlled by a syringe pump (SP210i Syringe Pump; WPI, Saratosa, FL, USA) and a multiport valve (either V-451; Upchurch Scientific, Oak Harbor, WA, USA, or VICI EHMA 12 port Microelectric Two-Position Actuator; Valco Instruments, Houston, TX, USA). Connections between the parts were bridged by Teflon tubing (0.3 mm inner diameter). A schematic of the setup is shown in Figure 2.

### The Particles

The migration experiments were performed with six different types of polystyrene microparticles, namely, green small, green medium, green large, red small, red medium, and red large, which were purchased from Microparticles GmbH. The particles are listed in Table 1. The particles were chosen such that two similarly sized particles of each color were measured to directly examine the impact of different coloring on acoustic mobility. The particle sizes were chosen such that the acoustic radiation force would dominate over any streaming forces. Any experiment was performed with a particle concentration of <5 × 10^6^ particles/mL suspended in Milli-Q water to avoid hydrodynamic particle–particle interactions (see Section 2 in the Supporting Information).

**Table 1:** List of Particles That Were Measured in This Study^a^

name	manufactured size (μm)	tag
PS-FluoGreen-5.0	5.19 ± 0.14	green small
PS-FluoGreen-8.0	7.81 ± 0.11	green medium
PS-FluoGreen-10.0	9.98 ± 0.11	green large
PS-FluoRed-5.0	4.99 ± 0.16	red small
PS-FluoRed-8.0	7.76 ± 0.12	red medium
PS-FluoRed-10.0	9.89 ± 0.11	red large

a Our fluorescent particles were manufactured by Microparticles GmbH (Microp.). Our measurements included six particle types, consisting of two fluorescent colors (red and green) and three different particle sizes for each color. The size ranges of the particle diameters as given by the manufacturer are added to the table.

**Figure 3:**
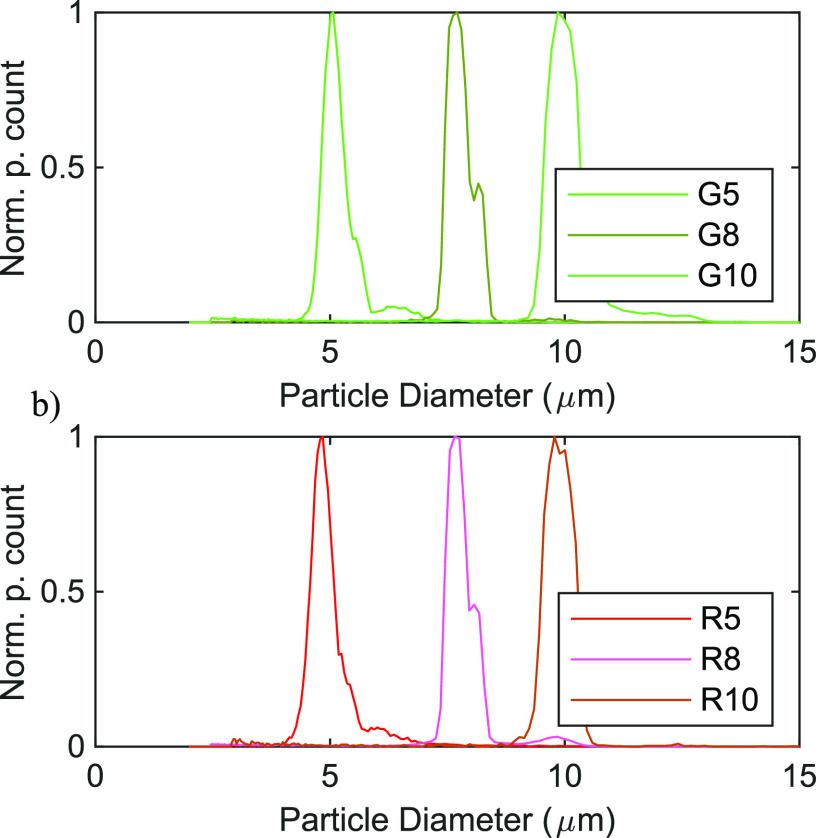
Normalized distributions of the particle diameters for the green particles (a) and red particles (b) measured by the Coulter counter. The normalization constants, i.e., the number of counts at the peak diameter, was 3521 for the green small, 1790 for the green medium, 9770 for the green large, 3833 for the red small, 19483 for the red medium, and 752 for the red large. The numerical values of the distributions are summarized in Table 2.

The acoustically induced particle migration velocity scales with the square of the particle radius and variations in particle size affected our measurements significantly. Therefore, the particles’ sizes were measured with a Coulter counter. The size distributions measured by the Coulter counter are shown in Figure 3 and summarized in Table 2.

**Table 2:** Coulter Counter Measurements of the Particle Diameters^a^

particle	count	mean (μm)	P16 (μm)	P84 (μm)
green small	36k	5.11	4.84	5.36
green medium	13k	7.78	7.54	8.07
green large	61k	9.98	9.72	10.25
red small	43k	4.86	4.58	5.13
red medium	118k	7.77	7.55	8.07
red large	5k	9.86	9.56	10.12

a The values were obtained from the distributions after removing the outliers. Count gives the number of particles that are included in the measurement. P16 and P84 denote the 16% and 84% percentiles, respectively.

### Theoretical Background

We considered a one-dimensional standing pressure wave in the *y*-direction and of amplitude *p*_*a*_, formally given by
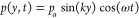
1where we used the angular frequency (ω), time (*t*), and wavenumber (*k*).

Balancing the Stokes’ drag and the acoustic radiation force according to Gorkov^6,7^ yielded the acoustophoretic migration velocity,
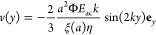
2
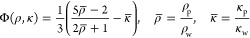
3
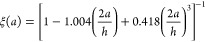
4where we used the particle radius (*a*), the acoustic contrast factor (Φ), the acoustic energy density (*E*_ac_), the fluid’s dynamic viscosity (η), the particle density (ρ_*p*_), the medium density (ρ_*w*_), the particle compressibility (κ_*p*_), the medium compressibility (κ_*w*_), and the unit vector along the *y* coordinate (**e**_*y*_). We also introduced the Faxén correction factor ξ(*a*)^19^ to the particle drag as a function of the particle radius *a* computed for our channel height *h* = 150 μm. The perpendicular correction factor from the sides of the channel was neglected as it only exceeded the parallel correction factor for ∼9% of our data points. Additionally, we defined the acoustic mobility mob_*i*_ of a species *i* by . Note that particles of positive acoustic contrast factor will collect in the pressure node.

Measuring the acoustic mobility, and thus the material properties, of an unknown particle by acoustic migration experiments usually involves two steps as both the acoustic energy density and the acoustic mobility appear as unknowns in eq 2. A common approach is to measure the acoustic energy density inside the channel by recording the trajectories of a known reference particle. In a second step, the acoustic mobility of the unknown particle can then be assessed by particle tracking experiments. This procedure is equivalent to assessing the acoustic mobility ratio between the reference and unknown particles.

### Migration Experiments

**Figure 4:**
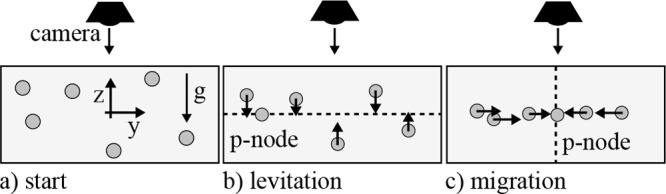
Schematic view of the channel cross-section during the particle tracking procedure: (a) the flow was stopped as soon as the suspended particles entered the field of sight, (b) the particles were acoustically levitated into the midheight (*z*-direction) of the channel, and (c) the particles were acoustically focused to the midwidth (*y*-direction) of the channel.

The migration experiments consisted of three steps, as shown in Figure 4. In the first step, illustrated in Figure 4a, the particles were suspended at a concentration of <5 × 10^6^ particles/mL and injected in the microfluidic device. As soon as the particles arrived in the field of sight the flow inside the channel was stopped by closing the multiport valve (Figure 4a). In a second step, the half-wavelength mode in the channel height was actuated at 4.82 MHz, which levitated the particles into the midheight of the channel (*z*-direction) against gravity, shown by Figure 4b and minimized the wall-induced drag forces. This step was not accounted for in the work by Hartono et al.^4^ Baasch et al.^5^ have shown that this can lead to errors of >6% in the estimated value of the cell compressibility. In our channel geometry, the Faxén correction factor, which corrects the Stokes drag coefficient for the added drag due to the top and bottom channel walls, yielded 1.035 and 1.072 for the 5- and 10-μm-diameter particles, respectively.

Additionally, the levitation also improved the particle tracking procedure as the focal plane of the camera could be set to match the nodal plane of the acoustic half-wave. In the third step, the migration step (Figure 4c), the levitation was turned off and the migration mode was excited at 1.97 MHz. This created a central acoustic nodal plane along the midwidth of the channel. The resulting trajectories of particles were recorded at a frame rate of 50 Hz. The procedure was repeated 20 times, rinsing and refilling the channel between each measurement.

### Measurement Procedure

In our experiments, the measurements were performed in three steps: first with the reference particles, then with the target particles under the exact same conditions, and finally with the reference particles again. This final measurement was included, so it was possible to confirm that the conditions did not change during the course of the experiment by comparing them with the initial reference particle measurements. In the first step, a series of images were collected as the reference particles migrated toward the pressure node. The velocity of the particles as a function of their positions was then extracted by particle tracking software (Defocustracker, by Barnkob and Rossi^20^). The sinusoidal reference curve was then fitted to the experimentally obtained particle velocities. More precisely, we used the parameters *A*, *B*, and *C* of the analytical approximation to the migration velocity ***v***(*y*) = *A* sin(*By* + *C*) as fitting parameters. The fitting parameters relate to the physical parameters from eq 2 via
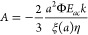
5and
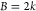
6

In the second step, the target particles were introduced into the channel, and the migration experiments as well as the data collection were repeated. Each mobility ratio data point was generated by dividing the velocity of a target particle *v*_tar_ at one position inside the channel by the fitted reference velocity at the same position *v*_ref_, which yielded
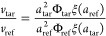
7The mobility ratio is then extracted via
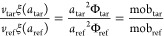
8

This allowed us to directly measure the acoustic mobility ratio between two particles by analyzing their migration velocities in an acoustic standing wave. One advantage of our method is that a small number of target data points is sufficient to measure the acoustic mobility ratio. Only the reference data points need to be sufficient to fit the sinusoidal reference curve. Each experiment was started and concluded by measuring the green small particles 20 times, which we used as reference particles.

### Filters and Compartmentalization

**Figure 5:**
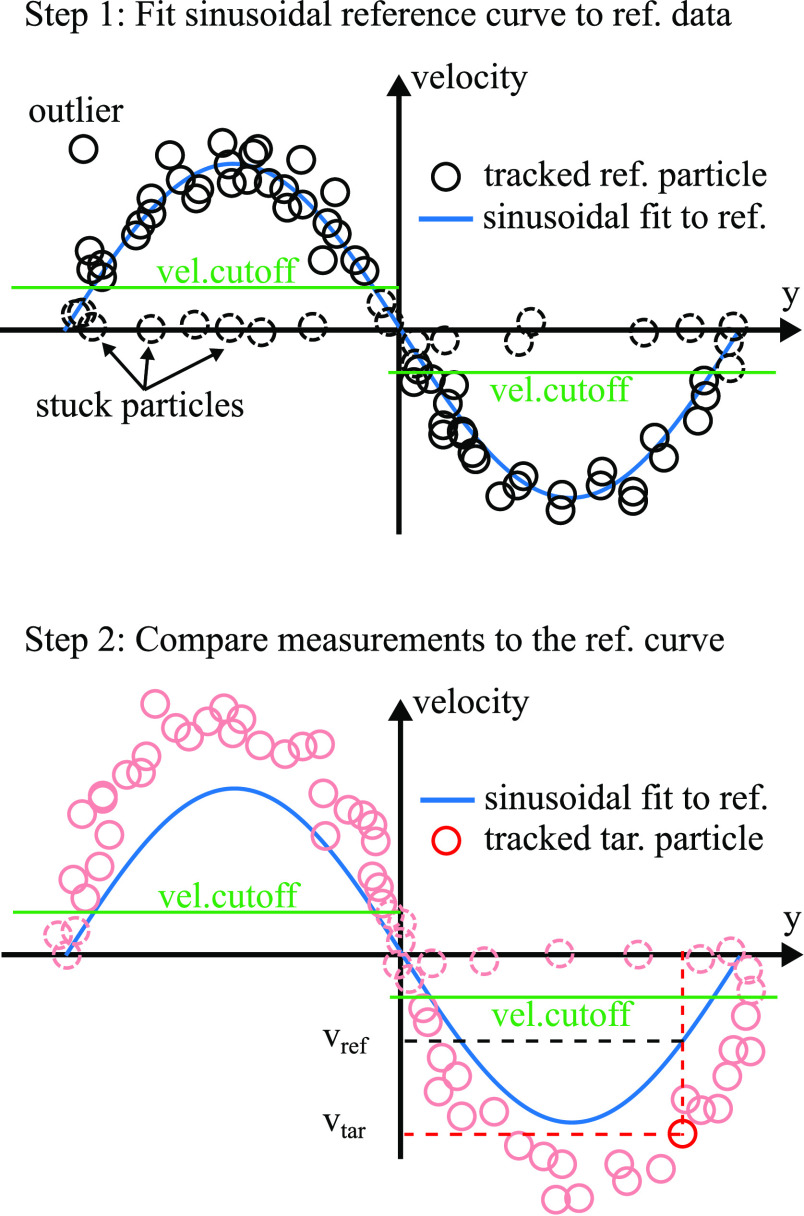
Measurement procedure consisted of two steps. First, the migration experiments were performed with the reference particles. A sinusoidal reference curve was then fitted to the velocity of the reference particles. In the second step, the velocity of the target particles was collected for each measured time step. The target particle’s velocity was then directly compared to the reference velocity using the sinusoidal reference curve. The ratio between the target particle’s velocity and the reference velocity was input into eq 8 with the Faxén correction to yield the mobility ratio for one data point.

The channel area of interest, in which we collected our results, was 650 μm long. Since the pressure amplitude can vary significantly over such a distance, we compartmentalized our area of interest into at least 8 equally long compartments (see Section 3 in the Supporting Information). The reference sinusoidal fit was then collected for each individual compartment. A velocity threshold was added to filter out the particles stuck to the channel walls, as shown in Figure 5. The velocity threshold was always chosen between 6.4 and 24 μm per second. We also removed outliers from the mobility ratio distribution of the target particles by removing data points that are further than three scaled mean absolute deviations from the median.

## Results and Discussion

**Figure 6:**
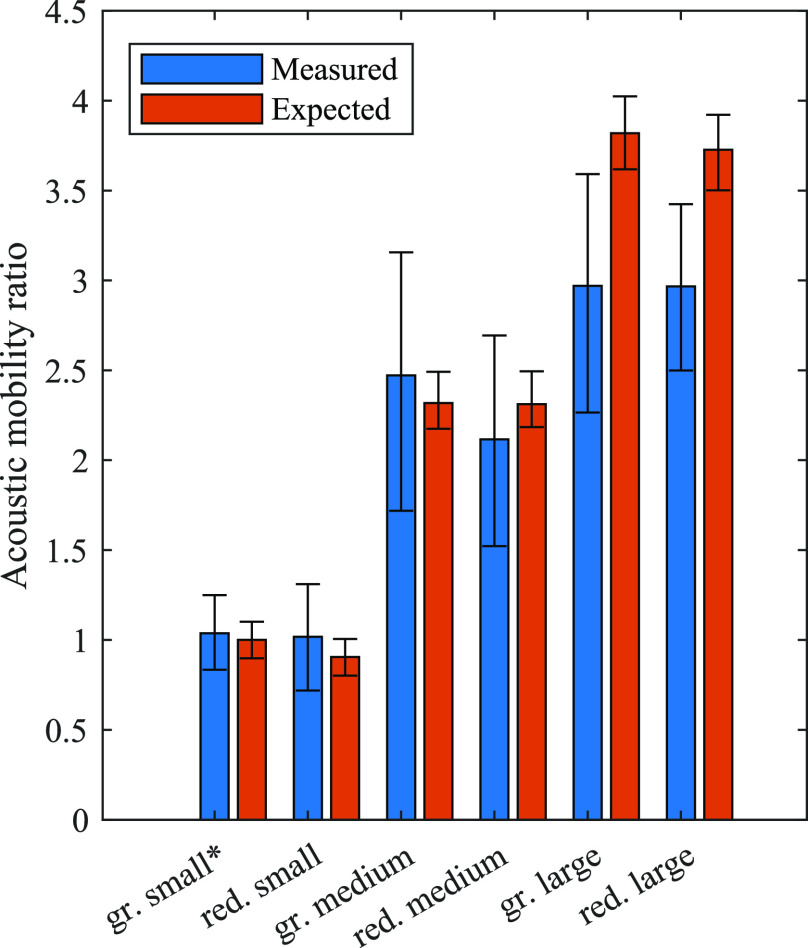
Summary of the measured and expected mobility ratios. The expected mobility ratios are based on the Coulter counter measurements of the particle diameters. The wings show the 16% and 84% percentiles of the data distributions and the height of the bars shows the mean value. The green small particle, indicated by an asterisk, was used as a reference particle for all the measurements.

The experiment was performed three times each for six different polystyrene particle types, consisting of three sizes of fluorescent green and red particles. All the resulting mobility ratios were given with respect to our reference particles (green small). The mean and standard deviations of the acoustic mobility ratio obtained from the migration experiments are shown in Table 3 and summarized in Figure 6. Each data point *i* for the expected acoustic mobility ratio Ψ_exp ,*i*_ was obtained from the mean radius of the reference particle *a*_mean,ref_ and one measured (Coulter counter) radius of a target particle *a*_*i*,tar_ by
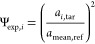
9The distribution, from which the mean values and percentiles were extracted, was then given by the set of all the data points. The mean values define the height of the bars in Figure 6 and the upper and lower bounds of the wings are given by the 16% and 84% percentiles. The 16% and 84% percentiles of our data are spread over ranges much larger than commonly reported in the literature. For example, Hartono et al.^4^ reported uncertainty intervals on the compressibility of ∼10% of the mean value. This small range was due to how they processed their data. They obtained one data point by fitting their measured trajectory data directly to theoretical predictions, which inherently averages the collected data points. Thus, their resulting acoustic mobility (and compressibility) distributions can be understood as a collection of means whose spread is more closely related to a confidence interval. The confidence intervals for our data are under 10% of the mean value in all our measurements.

**Table 3:** Measured Acoustic Mobility of Polystyrene Particles^a^

	Acoustic Mobility					
particle	measured	P16; P84	CI 95%	expected	P16; P84	# of data pts
green small	1.04	0.83; 1.25	1.035–1.04	1.00	0.90; 1.10	36k
green medium	2.47	1.72; 3.16	2.46–2.48	2.32	2.17; 2.49	27k
green large	2.97	2.27; 3.59	2.94–3.00	3.82	3.62; 4.02	1.9k
red small	1.02	0.72; 1.31	1.01–1.03	0.91	0.80; 1.01	4.4k
red medium	2.12	1.52; 2.69	2.10–2.14	2.31	2.18; 2.49	4k
red large	2.97	2.5; 3.42	2.95–2.98	3.73	3.5; 3.92	2.4k

a Every particle was measured with respect to the green small particles. We give the 16% (P16) and 84% (P84) percentiles for both the measured and the expected values, and the 95% confidence interval (CI 95%) on the measured mobility ratio.

We performed self-reference experiments to validate our method and to quantify the experimental errors. In the self-reference experiments the acoustic mobility ratio of the green small particles were assessed in reference to the same green small particles. The experiments showed a reasonable difference in the mean of 4%, which, as expected, lies well inside the spread (0.83–1.25) given by the percentiles. The measured mobility ratios for the green medium, red small, and red medium are in a reasonable range, compared to the expected value. In those cases, the deviations of the measured mean from the expected mean can be explained by errors in the measurement procedure and variations in the material properties. Interestingly, all the large particles (green large and red large) had a significantly lower (mean difference of 29% and 26% respectively) acoustic mobility ratio than expected. The difference in mean value and the small overlap in the percentile ranges could not be explained by errors due to our measurement technique, or from their size distribution. Thus, we concluded that the large particles have significantly different acoustic properties than the small particles. This indicates that care must be taken if polystyrene particles are used as a reference when performing quantitative measurements in acoustofluidic systems. Notably, even if the particles are made from the same material, they show a significant spread in their (acoustophoretic) properties.

### The Consequences for Cell Compressibility and Acoustic Energy Density Measurements

The uncertainty range of approximately ±30% in acoustic mobility ratio translates directly into acoustic energy density measurements. This means that without any further measurements on the acousto-mechanical properties of the polystyrene particles, a systematic error of up to 30% in acoustic energy density can be expected. When measuring the acoustic compressibility of cells via migration experiments, one usually first measures the acoustic energy density in the channel using a known reference particle. Then, in the next step, the acoustic mobility (Φ*a*^2^) of the cells is measured. From the acoustic mobility, the compressibility can be extracted if the cell and medium density, the cell radius, and the medium compressibility are known. Here, a percentage variation of up to 30% in the acoustic mobility measurements is to be expected. For the sake of simplicity, we assume that it is possible to measure the cell radius with absolute accuracy. Thus, the uncertainty range of the assessed cell contrast factor is ∼30%. This uncertainty on the contrast factor translates into measurements of the acoustic compressibility in the following way. Recall that the acoustic contrast factor is given by
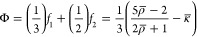
10Then, the variation of the particle compressibility δκ_P_, as a function of small variations of the acoustic contrast factor δΦ, is given by
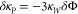
11

The percentual variation of the contrast factor (Φ_var_) is formally given by , or δΦ = Φ_var_Φ_0_, where Φ_0_ denotes the measured average of the contrast factor. Thus,
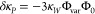
12The ranges that are obtained by applying eq 12 to the results given by Hartono^4^ are summarized in Table 4.

**Table 4:** Uncertainty Ranges for the Compressibility of the Biological Cells^a^

cell	density (kg/m^3^)	compressibility (1/TPa)	uncertainty range (1/TPa)
MCF-7	1068	422 ± 19	[408, 436]
HEPG2	1087	428 ± 12	[414, 442]
HT-29	1077	404 ± 16	[384, 424]
NIH/3T3	1079	378 ± 17	[349, 407]
MCF-12A	1068	377 ± 9	[350, 404]

a The compressibility and density values are taken from Hartono et al.,^4^ and the uncertainty ranges (uncty. rg.) are given by eq 12.

### Conclusion

We have measured the acoustophoretic properties of six different polystyrene particle types (two colors and three different sizes), with respect to fluorescent green polystyrene particles (Microparticles GmbH) 5 μm in diameter. For most particles, i.e., green small, red small, green medium, and red medium, we could not find any significant difference between the measured and expected mobility ratio. All the large particles, i.e., the green large and red large, showed a significantly lower acoustic mobility ratio with respect to our reference particle than what is expected from their sizes and material properties. This indicates that polystyrene particles can vary significantly in their acoustic properties, even though they are made from the same material. Thus, care must be taken if polystyrene particles are used as reference particles to characterize the acoustic energy density inside acoustofluidic manipulators, or if they are used to characterize the compressibility of cells or small organisms. The maximal difference of more than 25% in measured and expected mobility ratio can translate directly into acoustic energy density or cell property measurements and adds to their uncertainty ranges. The exact cause for this change in acoustic mobility is currently unclear, although a possible explanation might be the molecular weight and especially the length of the polymer. The polymer chain length may differ between larger and smaller particles, which could potentially impact their compressibility. All measurements have been performed with respect to the green small particles, since we have no means of confirming which particles have a compressibility and density closest to the values that were reported in the literature for polystyrene.^21−23^ Our main finding is that the particles present a large spread in their material properties with significant differences from the expected values, not only in the width of the distributions but also in their respective mean values.

## Supporting Information Available

S1: sketch of the Holder designed for the microfluidic device; S2: motivation and derivation for the maximal chosen experimental particle concentration in the paper; and S3, further explanation regarding the compartmentalization of the experimental data (PDF)
