# Supplementary material for: Acoustofluidic Properties of Polystyrene Microparticles
Source: Anal Chem. 2023 Jun 26;95(27):10346–52. doi: 10.1021/acs.analchem.3c01156 (PMC10339281; doi:10.1021/acs.analchem.3c01156)
Supplement: Supplementary file 1 — ac3c01156_si_001.pdf [file ac3c01156_si_001.pdf]

Acoustofluidic properties of polystyrene microparticles: Supporting Information

Alexander Edthofer,† Jakub Novotny,‡ Andreas Lenshof,† Thomas Laurell,† and Thierry Baasch*,†

†*Department of Biomedical Engineering, Lund University, 223 63 Lund, Sweden*

‡*Department of Bioanalytical Instrumentation, Institute of Analytical Chemistry of the CAS, 602 00 Brno, Czech Republic*

E-mail: thierry.baasch@bme.lth.se

Table of Contents

1. Drawings S2

2. Particle Concentration S2

3. Compartmentalisation S5

1. Drawings

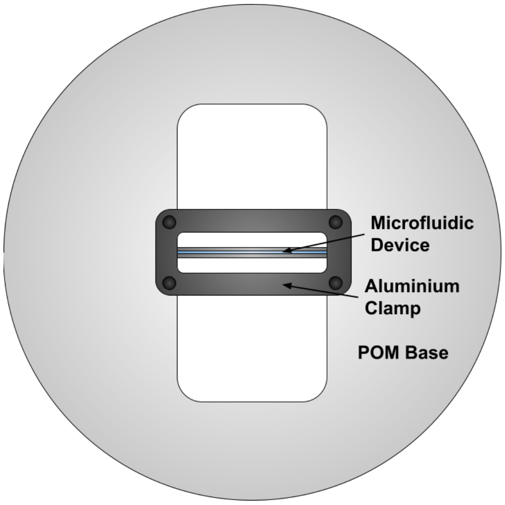

Figure S1: The acoustofluidic chip device and the holder that was used to fit it into the microscope.

2. Particle concentration

As the particles come close to each other, the hydrodynamic acoustic particle-particle interactions become more significant.[1,2] To neglect those interaction forces in measurements one needs to make sure that the particles have sufficiently large average distances. Here it is sufficient for the average inter-particle distance to be $\gg 4a$, as the hydrodynamic forces are negligible above this threshold.[1]

Let's assume that the particles are distributed in a 2-dimensional grid after the levitation, as illustrated in Fig. S2.

The total particle number N in the channel is described by the particle concentration C_{N} and the volume V,

$$N = C_{\mathrm{N}}V = C_{\mathrm{N}}wlh, \tag{1}$$

with $l \times w \times h$ being the dimensions of the channel. N can also be computed by their average

distance d and the dimensions of the plane:

$$N = \frac{wl}{d^2}. \tag{2}$$

Combining equations 1 and 2 yields

$$C_\mathrm{N} = \frac{1}{d^2 h}. \tag{3}$$

As d is the distance between the centers of two adjacent particles, the distance between their surfaces is $d - 2a$. Thus, for a distance between two particles of at least $4a$, d must be at least $6a$, giving us the following concentration for a critical particle concentration C_crit:

$$C_\mathrm{crit} = \frac{1}{36a^2 h}. \tag{4}$$

In this study, the largest particle has a diameter of 10 μm. As the height of the channel is 150 μm, this gives an expected critical concentration of 7.5 $\times 10^6$ particles/ml. As particles will not be evenly spread out, we assume that the actual critical concentration will be lower.

As an additional verification, we measured the acoustic energy density for particle concentrations of 10^6, $5 \cdot 10^6$, 10^7 and $5 \cdot 10^7$ particles/ml of 5.11 μm green fluorescent particles. The experiment was performed at three positions along the channel. The measured apparent acoustic energy density E_{ac} is displayed in Tab. S1. The experiment shows that the apparent acoustic energy density increases for particle concentrations larger than 10^7 particles/ml. This indicates the threshold after which the particles begin to interact hydrodynamically. To avoid these effects, all particle concentrations in the study were kept at $5 \cdot 10^6$ particles/ml or lower.

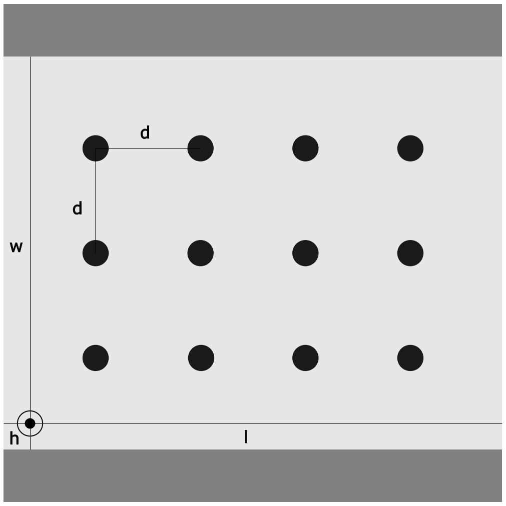

Figure S2: Assuming that the particles are evenly distributed in a plane after being levitated, the average distance d between the particles becomes a function of the dimensions of the channel and the particle concentration.

Table S1: The measured acoustic energy density depending on the particle concentration shows that the apparent acoustic energy density begins to increase above a threshold concentration between $5 \cdot 10^6$ and 10^7 Particles/ml.

Particle Concentration (N/ml)	$\mathrm{E_{ac}}(Pa)$ Position 1	$\mathrm{E_{ac}}(Pa)$ Position 2	$\mathrm{E_{ac}}(Pa)$ Position 3
10^6	3.40	7.30	9.80
$5 \cdot 10^6$	3.21	7.33	9.45
10^7	3.70	8.26	11.23
$5 \cdot 10^7$	5.00	10.14	14.12

3. Compartmentalisation

The acoustic energy field is not completely homogeneous along the channel due to the inherent three-dimensional nature of acoustic modes, as shown in Fig. S3. As the acoustic energy density is proportional to the acoustically induced particle velocity, these variations can cause significant variations when analyzing the trajectories of individual particles if the sinusoidal fit of the reference particles is averaged over a too broad part of the channel. To counter this, the recorded images were sliced into equally wide segments in the x-direction. The segments were less than 100 μm wide. The particles were assigned to a segment based on their average position in the x-direction. As the particle tracking was performed while the flow rate was 0, their movement in the x-direction was minimal and most particles would be contained within a single segment.

With this method, it was possible to measure the relative acoustic mobility of the particles on a more localized level, which increased the accuracy of the particle tracking.

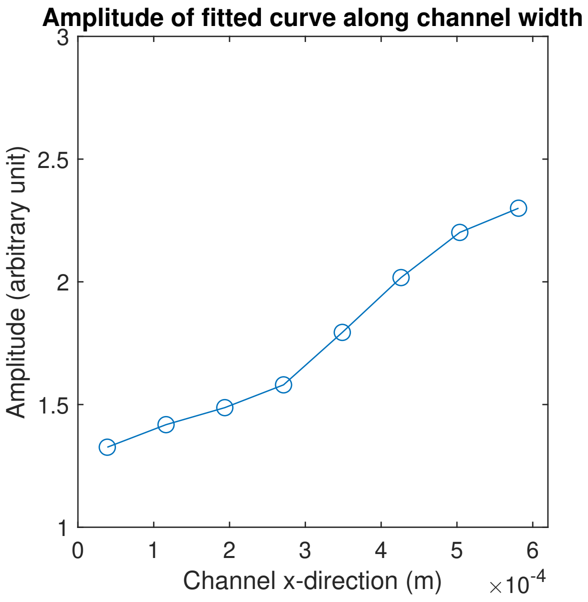

Figure S3: The amplitude of the sinusoidal fitting curve along the channel. The amplitude is proportional to the acoustic energy density and can clearly be observed to increase along the channel.

References

(1) Ley, M. W.; Bruus, H. Continuum modeling of hydrodynamic particle–particle interactions in microfluidic high-concentration suspensions. *Lab on a Chip* **2016**, *16*, 1178–1188.

(2) Baasch, T.; Leibacher, I.; Dual, J. Multibody dynamics in acoustophoresis. *The Journal of the Acoustical Society of America* **2017**, *141*, 1664–1674.
